# Desmoplastic small round cell tumor of the kidney: a case report

**DOI:** 10.1186/s13000-020-01015-w

**Published:** 2020-07-23

**Authors:** Dilek Ertoy Baydar, Ayse Armutlu, Oguz Aydin, Ayhan Dagdemir, Yarkin Kamil Yakupoglu

**Affiliations:** 1 Department of Pathology, Koc University School of Medicine, Topkapi, 34010 Istanbul, Turkey; 2 Department of Pathology, Ondokuz Mayis University School of Medicine, Samsun, Turkey; 3 Department of Pediatric Oncology, Ondokuz Mayis University School of Medicine, Samsun, Turkey; 4 Department of Urology, Ondokuz Mayis University School of Medicine, Samsun, Turkey

**Keywords:** Desmoplastic small round cell tumor, Kidney, WT1, EWSR1, Case report

## Abstract

**Background:**

Desmoplastic small round cell tumor (DSRCT) is a rare, aggressive neoplasm seen in children and young adults, usually manifested by involvement of abdominal serosa. Here, we present an unusual case of primary DSRCT of kidney.

**Case presentation:**

The patient was an 8-year-old girl with a large renal mass which was confused with primitive neuroectodermal tumor (PNET) in the needle biopsy. The tumor had a variegated histology revealing frequent pseudo-rosette formations, pseudopapillary architecture, rhabdoid, clear or pleomorphic cells in addition to typical small round cell morphology and desmoplasia. It showed immunohistochemical features of DSRCT, and *EWSR1* re-arrangement.

**Conclusions:**

Proffering this diagnosis is particularly difficult for tumors of viscera because of the incognizance of the entity in these locations. Moreover, DSRCT is a great mimicker and may get easily confused with more common kidney malignancies of childhood such as Wilms tumor, PNET/EWS, rhabdoid tumor, clear cell sarcoma, and other small round cell tumors as well as renal cell carcinomas. The distinction is critical as the accurate therapeutic approach will require correct diagnosis.

## Background

Desmoplastic small round cell tumor (DSRCT) is a rare, distinct entity that was first described by Gerald and Rosai in 1989 [1]. Predilection for adolescent males, predominant intraabdominal location involving serosal surfaces, nesting pattern of growth, focal rhabdoid morphology, prominent desmoplastic reaction, immunohistochemical reactivity for epithelial, neural and muscle markers, and highly aggressive clinical behavior are its main features. DSRCT shows a specific reciprocal chromosomal translocation, t(11;22)(p13;q12) (*EWSR1-WT1* fusion) which generates a chimerical protein with transcriptional regulatory activity. Extraserosal DSRCTs are extremely rare with only a few cases reported in lungs, ovary, soft tissues, bones, intracranial and sinonasal locations [2, 3].

DSRCT primary of the kidney was first described by Su, et al. [4] in 2004 and since then only a total of 12 cases have been reported in the literature (Table 1) [5–12]. Herein, we present the thirteenth case of renal DSRCT that had variant histological features mimicking various types of other neoplasia. The pathologic diagnosis of this entity can be markedly challenging when it develops in visceral organs such as kidney and especially if diverse and confounding microscopic features are present.

**Table 1:** Clinicopathologic features of the cases described in the literature (^a^)

	Age / Sex	Clinical presentation	R / L side	Gross Findings	Microscopic Findings	Immunohistochemistry	Molecular pathology	Therapy	Follow-up	Publication
**Case 1**	41 / F	Incidental	Left	Renal mass at hilar region, 6x5x5 cm, invaded into perirenal soft tissue	Nests of small round blue cells within desmoplastic stroma; occasional rosette-like formations; necrosis and numerous mitotic figures	CK (AE1/AE3) (+), Desmin (+), Vimentin (+), NSE (+), EMA (−), CK7 and 20 (−), CD99 (−), S100 (−), Chromogranin (−), Synaptophysin (−)	RT-PCR showing *EWSR1-WT1* fusion	Surgery	Alive (NED) at 18th mo.	Su, et al., [4]
**Case 2**	7 / F	Gross hematuria following a fall	Left	Confined within renal capsule, 3.7 × 3.7 × 3.2 cm	Small round undifferentiated cells, necrosis and epithelioid component	Desmin (+), WT-1 (+), CD99 (+), SMA (+), EMA (+), Myogenin (−)	RT-PCR showing *EWSR1-WT1* fusion	Surgery + CT	Alive (NED) at 12th mo.	Eaton, et al., [5]
**Case 3**^**b**^	6 / F	Renal mass (no details about clinical presentations)	Left	3.7 cm mass confined to kidney	Nests, sheets or cords of small undifferentiated cells; numerous mitotic figures, no desmoplasia	CK (+), Desmin (+), Vimentin (+), WT1 (+), FLI-1 (+), CD56 (+); EMA (−), CD99 (−), Myogenin (−), S100 (−), Chromogranin (−), Synaptophysin (−)	Dual color FISH showing *EWSR1-WT1* translocation and RT-PCR showing *EWSR1-WT1 *fusion	Neoadjuvant CT + Surgery	Alive (NED) 24th mo.	Egloff, et al., [6] and Wang, et al., [7]
**Case 4**	6 / F		Left	13.4 cm mass showing renal sinus invasion		CK (+), EMA (+), Desmin (+), Vimentin (+), CD99 (+), WT1 (+), FLI-1 (+), CD56 (+), Myogenin (−), S100 (−), Chromogranin (−), Synaptophysin (−)		Surgery + CT	Pulmonary metastasis at 32nd mo. CT, stem cell transplantation. NED a year later	Wang, et al., [7]
**Case 5**	6 / F		Left	9 cm mass with perirenal soft tissue and renal sinus invasion		CK (+), EMA (+), Desmin (+), Vimentin (+), CD99 (+), WT1 (+), FLI-1 (+), CD56 (+), Myogenin (−), S100 (−), Chromogranin (−), Synaptophysin (−)			Alive (NED) at 22nd mo	
**Case 6**	8 / M		Left	9.2 cm mass with renal sinus invasion		EMA (+), Desmin (+), Vimentin (+), CD99 (+), WT1 (+), FLI-1 (+), CD56 (+), CK (−), Myogenin (−), S100 (−), Chromogranin (−), Synaptophysin (−)			Intraabdomimal recurrence and liver metastasis at 20th mo. CT. AWD	
**Case 7**	14 / F	Gross hematuria, fever and self-disvovered abdominal mass	Left	17.5x12x11 cm mass invading renal sinus and perinephric fat, and focally extending to Gerota’s fascia; metastatic lymph node in hilar region	Small ovoid-spindle blue cells in solid sheets and large nests; rare rosette-like structures; numerous mitotic figures; tumor thrombi in perinephric blood vessels	EMA (+), Desmin (+), Vimentin (+), WT1 (+), CD56 (+), Chromogranin (focal +), Synaptophysin (rare +), CK (AE1/AE3) (−), CD99 (−), S100 (−)	RT-PCR showing *EWSR1-WT1 *fusion	Surgery + CT + local RT	Liver and lung metastases at 8th mo.	Collardeau-Frachon, et al., [8]
**Case 8**	10 / M	Gross hematuria, abdominal pain, palpable mass	Right	14 × 11 cm mass	Small blue round cells with desmoplasia; occasional rosette-like formations.	CK (+), Desmin (+), CD99 (−), WT1 (+), FLI-1 (+); S100 (−), Chromogranin (−), Synaptophysin (−)	RT-PCR showing *EWSR1-WT1* fusion	Surgery + CT + local RT	Liver, lung, bone, lymph node metastases. AWD at 12th mo	da Silva, et al., [9]
**Case 9**	20 / M	Renal mass and pulmonary nodules (no details about clinical presentations)	Right	8 cm mass with areas of hemorrhage and necrosis, invading renal vein grossly	Elongated to round cells with scant cytoplasm in sheets and occasionally a vague nodular pattern, frequent mitotic activity, lacked prominent desmoplasia	CK (+), Desmin (+), Vimentin (+), CD56 (+); WT1 (cytoplasmic +), CD99 (−), MyoD1 (−), NSE (−), RCC Ag (−), EMA (−), Myogenin (−), S100 (−)	FISH showing *EWSR11* rearrangement and RT-PCR showing *EWSR1-WT1* fusion	Surgery	Pulmonary metastases at presentation, local recurrence after surgery. Exitus at 2nd year	Rao, et al., [10]
**Case 10**	7 / M	Gross hematuria, microscopic hematuria and intermittent back pain 3 years priorly	Left	Polypoid mass confined to the renal collecting system, extending into proximal and mid ureter, no involvement of renal parenchyma	Spindled and polygonal tumor cells, rare rosettes, low mitotic rate	CD99 (+), Vimentin (+), Desmin (focal+), Actin (focal+), WT1 (focal+), PAX2 (+); PAX8 (−)	FISH showing *EWSR1* rearrangement, karyotyping showing t(11;22) (p13;q12).	Surgery + CT + RT	Alive (NED) (duration unknown)	Eklund et al., [11]
**Case 11**	6 / M	Facial swelling and pain, headache, decreased oral intake	Right	5.7 × 5.5 × 4.7 cm mass with large areas of central necrosis, invading hilar soft tissues	Sheets of poorly differentiated round cells, no desmoplastic stroma	Bcl-2 (+), CD99 (+), desmin (+), vimentin (+), CD56 (+), and FLI-1 (+), WT1 (−), Synaptophysin (−), SMA (−), Myogenin (−), Myo-D1 (−), CD31 (−), CD34 (−), Napsin (−)	RT-PCR showing *EWSR1-WT1 *fusion	No information	Metastatic disease (multiple, bone and lungs) at presentation. No further follow-up information	Walton, et al., [12]
**Case 12**	8 / F	Abdominal pain	Left	11x9x7 cm mass with renal pelvis, perirenal fat tissue and adrenal gland invasion	Nests, cords, sheets of small round cells within desmoplastic stroma; frequent rosette-like structures, psedopapillary appearance and frequent rhabdoid cells.	CK (+), EMA (+), Desmin (+), Vimentin (+), CD56 (+), WT1 (+), CD99 (−), Bcl-2 (−), MUC4 (−), Myogenin (−), Myo-D1 (−), SMA (−), S100 (−), Chromogranin (−), Synaptophysin (−)	FISH showing *EWSR1* gene re-arrangement	Neoadjuvant CT + surgery + adjuvant CT	Multiple metastases (liver, lungs and lymph nodes). Exitus at 30th mo.	Current case

*NED* No evidence of disease, *AWD* Alive with disease, *CT* Chemotherapy, *RT* Radiotherapy

^a^Table does not include a case reported by Janssens E, et al. (2009) [13] as the article could not be reached by any means

^b^Case 3 was first reported by Egloff, et al. (2005) [6] and also included among 4 patients in the case series published a year later by Wang, et al. (2007) [7]

## Case presentation

### Clinical history

An 8-year-old girl complained of abdominal pain and an ultrasonography found a large mass in her left kidney. Abdomimal MRI showed that it was a heterogenous lobulated solid lesion measuring 80x92x118 mm in size with cystic and necrotic areas. Needle biopsy from the tumor was diagnosed in an outside center as a small round blue cell tumor consistent with PNET/EWS. The patient had multiple lung, liver, adrenal and lymph node metastases at initial presentation. After 6 cycles of neoadjuvant chemotherapy, left radical nephrectomy was performed. Macroscopic examination showed 11x9x7 cm grey-white solid mass that occupied most of the organ parenchyma, invading also renal pelvis, perirenal soft tissue and adrenal gland extensively. Paraffin blocks of both needle biopsy and nephrectomy material were sent to our institution for consultation.

### Pathology

**Fig. 1:**
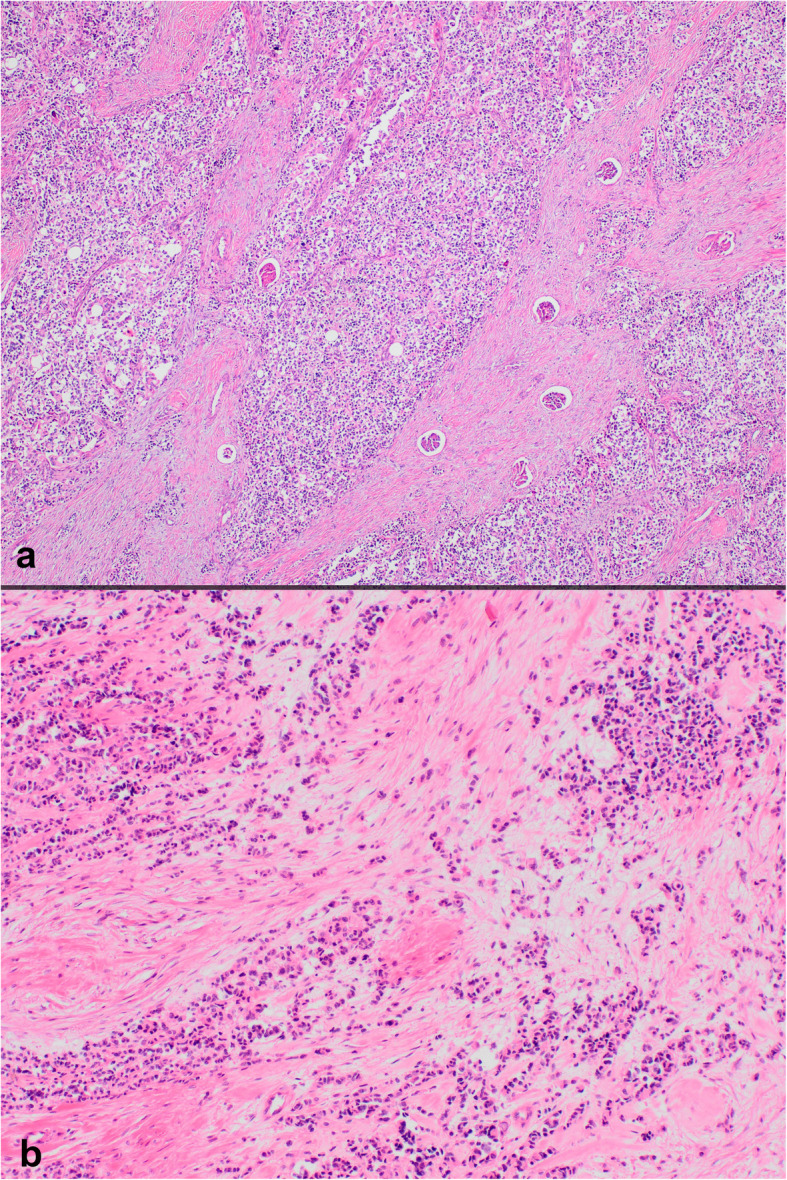
Sheets, nests and cords of neoplastic cells in a desmoplastic stroma (**a** H&E × 40; **b** H&E × 100)

**Fig. 2:**
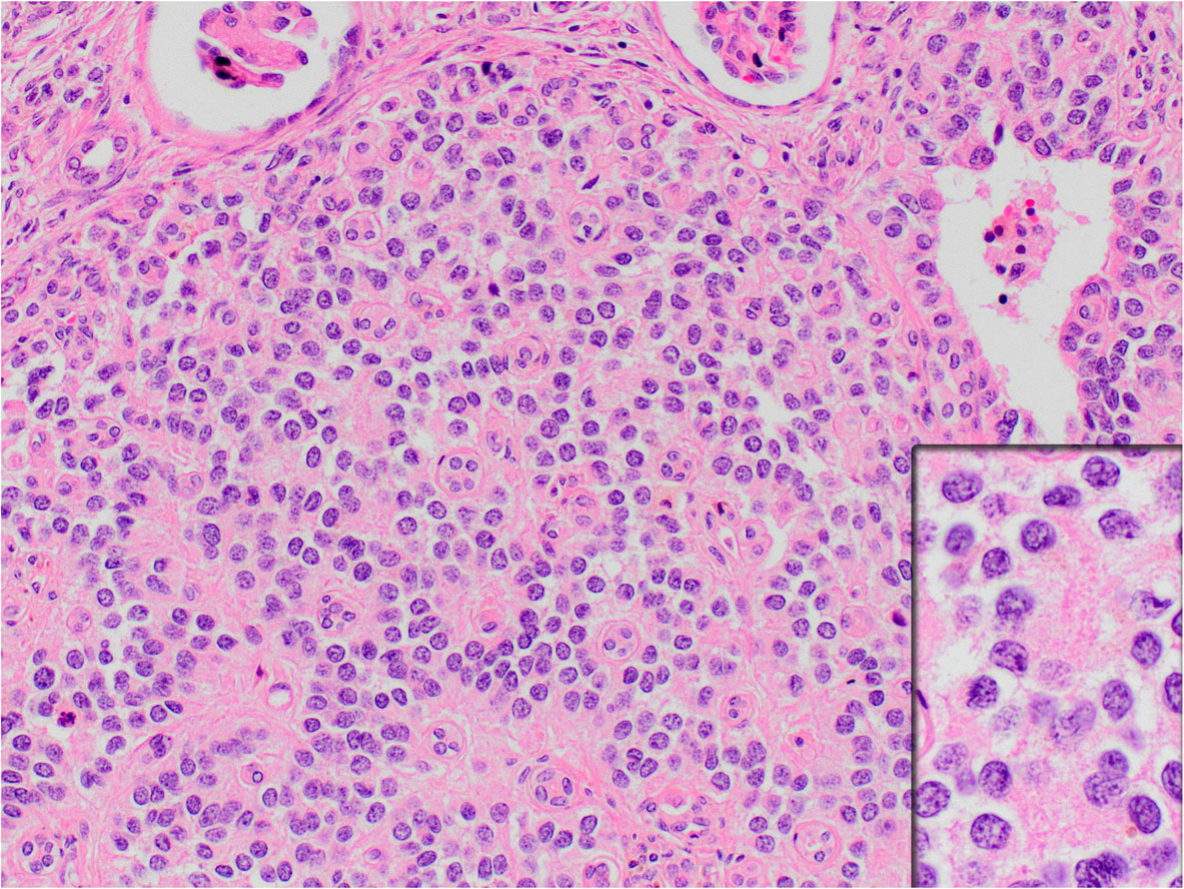
Rosette or tubule-like formations (H&E × 200; inset*:* H&E × 400)

**Fig. 3:**
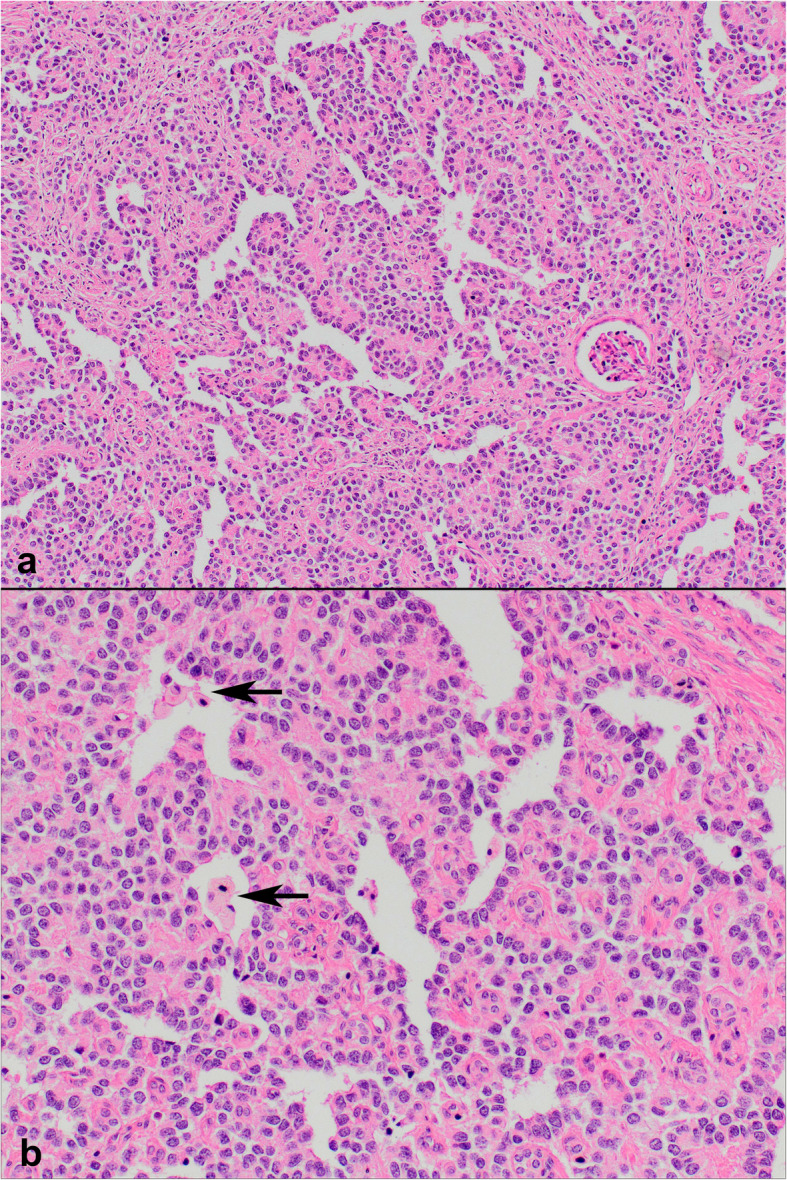
Pseudo-papillary pattern with foamy histiocytes (arrows) (**a** H&E × 100; **b** H&E × 200)

**Fig. 4:**
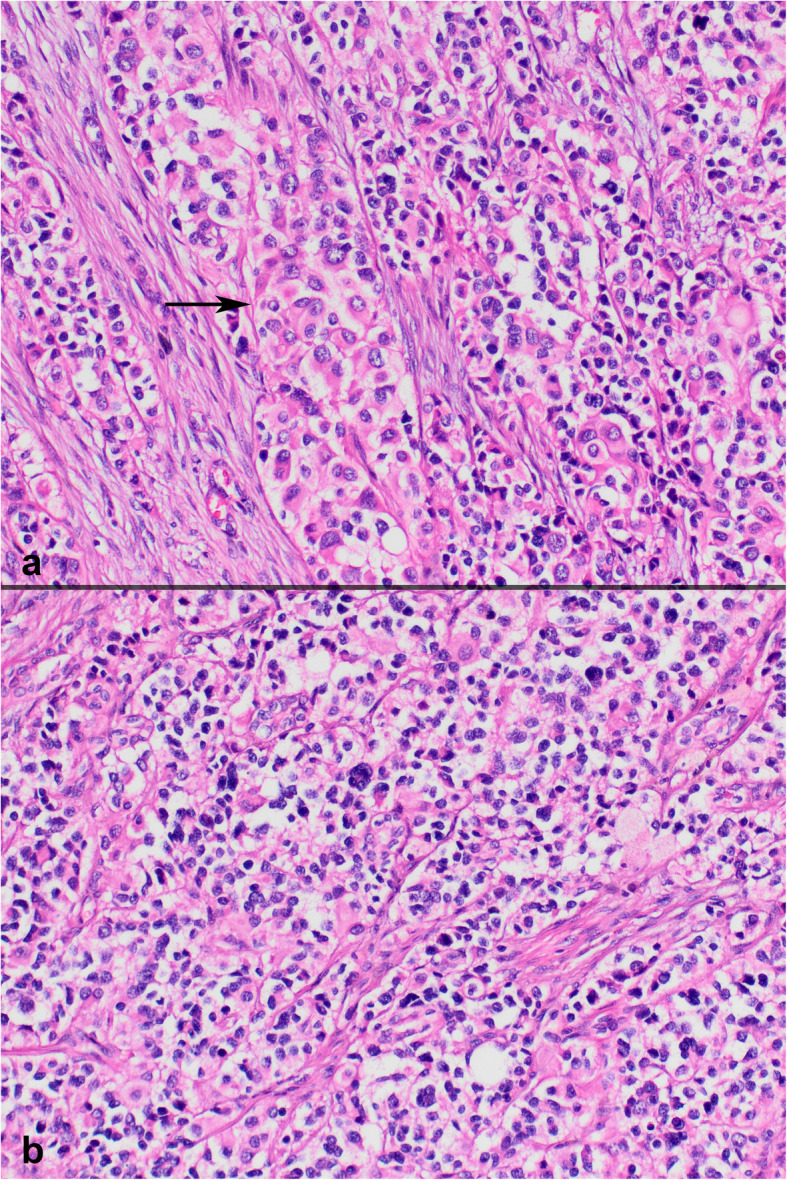
Rhabdoid cells (pointed by an arrow) (**a**) and cells with clear cytoplasm or pleomorphic nuclei (**b**) (**a** H&E × 200; **b** H&E × 200)

**Fig. 5:**
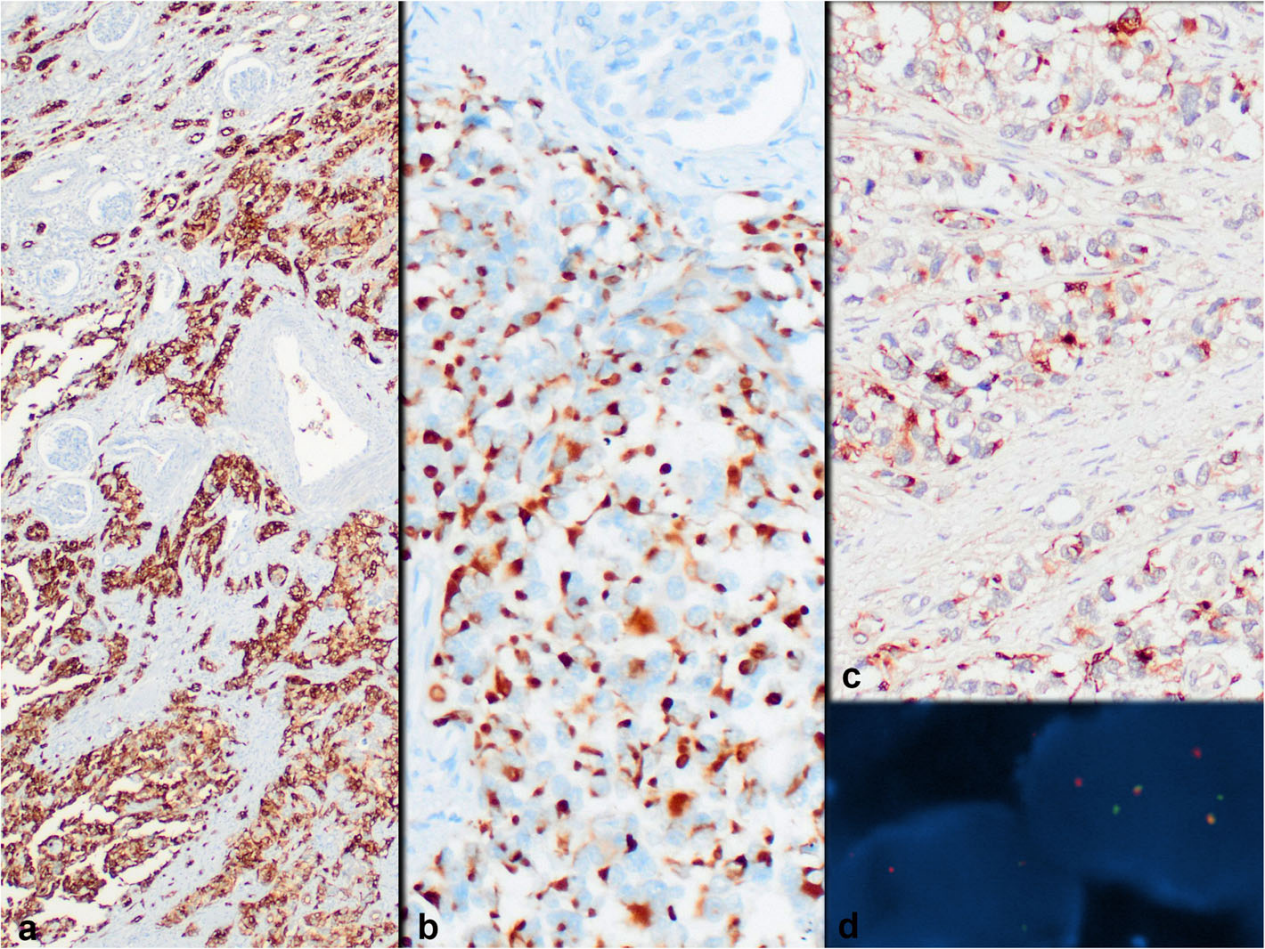
Strong EMA (**a**), perinuclear dot-like desmin (**b**) and non-specific cytoplasmic (non-nuclear) WT1 expression (**c**) by neoplastic cells, (**d**) is FISH analysis showing *ESWR1* rearrangement (**a** Immunohistochemistry, anti-EMA Ab × 40; **b** Immunohistochemistry, anti-desmin Ab × 200; **c** Immunohistochemistry, anti-WT1 (N-terminal) Ab × 200; **d** Dual Color Break Apart specific locus FISH probe targeting *EWSR1* gene at 22q12.2 chromosomal region; green and red signals mark the 5′ and 3′ ends of the gene respectively)

On histopathologic examination, neoplastic cells formed nests, cords and sheets within desmoplastic stroma (Fig. 1). Tumor also revealed intermittent areas of primitive tubule or rosette-like structures (Fig. 2). Furthermore, occasional foci appeared to have (psedo) papillary architecture with foamy histiocytes which was possibly due to drop-outs and loss of cohesion between cells (Fig. 3). Most neoplastic cells were small with narrow cytoplasm and round monotonous hyperchromatic nuclei. However, there were areas that contained unusually large amounts of eosinophilic, clear or vacuolated cytoplasm. Some cells revealed rhabdoid features, or pleomorphic, even multilobated nuclei (Fig. 4). Immunohistochemically, neoplasm diffusely expressed EMA, pan-cytokeratin, CD56, vimentin and desmin (paranuclear dot-like) whereas it stained negative for synaptophysin, chromogranin, S100, CD99, bcl-2, myo-D1, GATA3 and PAX8 (Fig. 5a-b). Nuclear INI1 was intact. Antibodies directed to N-terminus of WT1 protein stained cytoplasm of the tumor cells non-specifically without nuclear immunoreactivity (Fig. 5c). FISH analysis with a break-apart probe proved *EWSR1* gene re-arrangement in the neoplastic cells (Fig. 5d). Our final diagnosis was desmoplastic small round cell tumor of the kidney.

### Follow-up

The child was given multiple cycles and different combinations of adjuvant chemotherapy. She had a local relapse in the 2nd year that underwent salvage resection. Chemotherapy was continued thereafter, but she died in the 30th month from her first operation due to disease progression and wide-spread metastases.

Informed consent was obtained from the parents of the deceased child for the publication of the patient information and microscopic images.

## Discussion and conclusions

DSRCT is a rare, aggressive sarcoma placed in the family of small round cell tumors, typically seen in children and young adults with a male predilection. The disease most commonly originates in the abdominal or pelvic serosa. Primary DSRCT in extraserosal sites is extremely unusual. Classical histology of DSCRT is the nesting pattern of small round to oval cells separated by prominent desmoplastic stroma, focal rhabdoid features, and immunohistochemical profile with peculiar coexpression of epithelial (EMA and keratin), neural (NSE) and mesenchymal (vimentin and desmin) markers. The cytogenetics of a case of DSRCT, featuring a diploid DNA content and t(11;22)(p13;q12) was first reported in 1992 by Sawyer JR, et al. [14] In1994, Ladanyi and Gerald defined the consistent fusion between *EWSR1* and *WT1* genes in DSRCTs [15]. In a publication in 1995, they proved that DSRCT represents the third tumor type associated with *EWSR1* translocation, and it is the only malignancy holding *EWSR1* - *WT1* rearrangement [16].

Urogenital DSRCT may involve bladder, ureters, prostate and paratesticular structures [17]. Primary DSRCT of the kidney was first noted by Su, et al. [4] in 2004 and 12 cases have been reported in the literature thereafter [5–12] albeit one of them has an inaccessible publication. Although male predilection is emphasized in abdominal DSRCTs, most of renal cases including ours have been female (F/M = 7/5). Majority were detected incidentally or presented with gross hematuria and/or abdominal pain. Most tumors had infiltrative appearance with a size ranging from 3.7 to 17.5 cm in diameter. Two cases were confined to kidney [5, 7] and one case was limited to the renal collecting system without parenchymal involvement [12]. Interestingly, 9 out of 12 renal DSRCTs preferred left kidney.

DSRCTs may manifest unusual histologies such as tubule or rosette-like structures, papillary formations, abundant rhabdoid cells, predominantly spindle cell morphology, ample clear cytoplasm and absence of significant desmoplasia. The recognition of the morphologic diversity is important to avoid a misinterpretation during pathologic diagnosis especially when tumors are located in unusual sites. DSRCT of kidney appears to be a childhood neoplasm as almost all cases were pediatric, 6 to 8 years being the most frequent, with only two adult patients. Given this and also overlapping histopathologic features, main differential diagnosis of DSRCT is Wilms tumor (WT) in the kidney, the most common renal malignancy of children. Although the peak incidence of Wilms tumor is between 2 and 3 years, it can occasionally occur at later ages. Blastemal-predominant WT can be challenging to distinguish histologically from DSRCT and pseudo-rosette or tubule-like arrangements in DSRCT may mimic epithelioid component of WT. DSRCT localized to the kidney can lack desmoplastic reaction or WT can demonstrate desmoplasia. Both tumors show nuclear positivity immunohistochemically with antibodies against carboxy terminus of WT1 protein. Yet, immunohistochemistry is still useful in the discrimination: While WT is characterized by dual nuclear immunoreactivity for both amino- and carboxy-terminus WT1 antibodies, neoplastic nuclei in DSRCT do not stain with antibody recognizing amino-terminal of WT1 although non-specific cytoplasmic positivity can be seen as in our case. This can be explained by the fusion of the *EWSR1* gene to the last three exons (carboxy-terminus) of *WT1* in DSRCT. The re-arrangement produces a protein containing the zinc finger region of WT1 which needs C-terminal antibodies for recognition. It has been shown that blastemal WT may express paranuclear desmin in 50% of cases [18], however this is usually not widespread. Extensive (> 75%) paranuclear dotlike desmin positivity in addition to negative PAX8 and bland nuclear features will suggest DSRCT [18].

Other pediatric renal tumors, clear cell sarcoma and rhabdoid tumor, may also need to excluded from DSRCT in the kidney although these two preferentially occur in infancy or at very early childhood. DSRCT lacks capillary network of clear cell sarcoma and large nucleolus or prominent cytoplasmic inclusions of rhabdoid tumor. It has intact nuclear INI1 protein expression contrary to rhabdoid tumor. Clear cell sarcomas are negative for epithelial and muscle markers, and they have recently been shown to overexpress nuclear BCOR protein [19, 20].

DSRCT may mimic PNET/EWS, especially when it has a solid growth pattern. PNET/EWS was the first diagnosis in our patient, given to the needle biopsy taken from the mass, which was mainly suggested by the presence of frequent pseudorosettes in the tumor. These two different tumor types have similar age distribution, similar cytology and both harbor *EWSR1* rearrangements. Keratin expression may be seen nearly in 25% of Ewing sarcomas, but desmin positivity is exceedingly rare and keratin plus desmin coexpressing Ewing sarcoma has not been asserted. DSRCTs show more variable expression of CD99, rather than the diffuse membranous positivity typical of Ewing sarcoma. The characteristic translocation of Ewing sarcoma involves *EWSR1* and the ETS family of transcription factors, not *WT1*, and it lacks nuclear WT1 expression. The break-apart FISH assay for *EWSR1* will not be helpful in the differential diagnosis between DSRCT and PNET/EWS as one fusion partner in both tumors is this same gene. Given the previous reports of a few curious cases carrying hybrid features of both DSRCT and PNET/EWS but with *EWSR1-FLI1* or *EWSR1-ERG* fusion [21, 22], the gold standard for the definitive diagnosis of DSRCT would be demonstration of the *EWSR1-WT1* fusion by RT-PCR when feasible. It was not possible in our case due to low quality of extracted RNA from paraffin block.

Lymphoma/leukemia, metastatic neuroblastoma, poorly differentiated synovial sarcoma and rhabdomyosarcoma are the other tumors that need to be considered in the differential diagnosis of renal DSRCT. Lymphoma/leukemia often demonstrate a diffuse growth pattern and do not exhibit the cohesion and nuclear features of DSRCT, and can be excluded by a panel of lymphoid markers or TdT. Neuroblastoma occurs in very young children, over 90% being diagnosed below 5 years of age. Clinical and laboratory evaluation will usually reveal an adrenal mass and elevated catecholamine metabolites in urine. Neuroblastoma lacks the specific chromosomal translocation and all show HISL-19 expression. Synovial sarcoma characteristically harbors SYT-SSX gene fusion (t(X;18)(p11;q11)). Rhabdomyosarcoma generally does not have a desmoplastic stroma and unlike DSRCT, it will express myogenin and MyoD1, and the majority of alveolar rhabdomyosarcoma have FOXO1 fusions.

In our case, there were areas of cellular discohesion with groups of foamy histiocytes, leading to focal pseudopapillary architecture and bringing papillary type renal cell carcinoma (RCC) into consideration. Additionally, we have observed some small nests consisted of neoplastic cells with clear cytoplasm, reminiscent of clear cell RCC. Strong cytokeratin and EMA expression might favor an epithelial neoplasm, however negative immunoreactivity for PAX8 turned us away from the renal cell origin in the first round.

DSRCT is known to have a poor prognosis. Our patient who presented with multiple distant metastases at the initial diagnosis died at the 30th month despite radical operation and intensive chemotherapy. However, the detection of the disease at early stage and complete resectability may provide significant prognostic benefit as previously reported: 6 out of 11 renal DSRCTs were stated alive without disease, keeping in mind that the follow-up durations are too short to drive a reliable conclusion. The best therapeutic modality has yet to be explored for renal DSRCT. A combination of total resection and chemotherapy seems to be the most preferred strategy at the moment.

As a conclusion, DSRCT is a rare disease, but should be considered in the differential diagnosis of small round cell tumors of the kidney in pediatric patients. This is important as each one of those tumors has different clinical behavior, prognosis, and treatment implications. Immunohistochemical and molecular studies have particular guidance for the right analytic approach, and documentation of *EWSR1-WT1* fusion is the “gold standard” for the diagnosis of DSRCT as it appears exceedingly characteristic for this disease.

## Data Availability

Stained and unstained slides of the case can be provided if required.

## Abbreviations

DSRCT: Desmoplastic small round cell tumor
PNET: Primitive neuroectodermal tumor
EWS: Ewing sarcoma
WT: Wilms tumor
RT-PCR: *Reverse transcription polymerase chain reaction*
